# Supplementary material for: A feature transferring workflow between data-poor compounds in various tasks
Source: PLoS One. 2022 Mar 30;17(3):e0266088. doi: 10.1371/journal.pone.0266088 (PMC8967016; doi:10.1371/journal.pone.0266088)
Supplement: Attachment — Submitted filename: Response to Reviewers.docx [file pone.0266088.s001.docx]

Dear academic editor and reviewers,

I am very appreciated for your comments and suggestions. I have revised this manuscript and especially paid much attention to your comments and suggestions. The details of the revision are as follows:

Replies to the academic editor:

1. The style of the manuscript has been modified in the latex file to comply with the requirements of the template.

2. The completeness and style of the reference list have been reviewed, and the retracted articles are not cited.

3. The statement “There was no additional external funding received for this study.” has been included in our updated Funding Statement.

4.“The funder provided support in the form of salaries for authors [XS, JZ, BC, HY and HX], but did not have any additional role in the study design, data collection and analysis, decision to publish, or preparation of the manuscript. The specific roles of these authors are articulated in the ‘author contributions’ section.” has been included in our updated Funding Statement.

5. "This does not alter our adherence to PLOS ONE policies on sharing data and materials.” has been included within our Competing Interests Statement.

6. An updated Funding Statement and Competing Interests Statement have been included in our cover letter.

Replies to Reviewer #1:

1. The comparison tables (Tables 1 and 2) for exiting terms and proposed idea with all the parameters has been added in the results.

Replies to Reviewer #2:

1. The novelty and motivation of the proposed method have been highlighted in the abstract as: Owing to an overall insufficiency and imbalance of drug data, it is hard to accurately predict drug activity and toxicity of multiple tasks by the existing models. To solve this problem, this paper proposed a two-stage transfer learning workflow to develop a novel prediction model, which can accurately predict drug activity and toxicity of the targets with insufficient observations.

2. The challenges and limitations of the proposed work have been highlighted in the conclusion as: In order to make the model proposed in this paper can be used in common situation, it is essential to establish massive multi-task datasets for pretraining, which is also an interesting attempt for our future research work.

3. Some statistical analysis tests, as illustrated in Figures 9, 10 and 11, have been added to validate the results.

4. The manuscript has been proofread to eliminate typos errors. For example, line 21 on page 2 has been revised to read: For example, due to the difficulty of obtaining tumor tissue fragments of bone metastasis, the scarcity of sarcomatous-type tumors, and the difficulty in culturing bone tissue into cell lines, the number of cell models is insufficient.

We thank you in advance for considering our manuscript for publication in PLOS ONE.

Sincerely,

Xiaofei Sun
